# Hybrid Amino Acid Ligand-Regulated Excited Dynamics of Highly Luminescent Perovskite Quantum Dots for Bright White Light-Emitting Diodes

**DOI:** 10.3390/nano14151266

**Published:** 2024-07-29

**Authors:** Baoye Hu, Weiqiang Zhang, Ya Chu

**Affiliations:** 1 School of Physics Science and Information Technology, Liaocheng University, Liaocheng 252059, China; 2320110508@stu.lcu.edu.cn; 2 College of Chemistry and Chemical Engineering, Liaocheng University, Liaocheng 252059, China

**Keywords:** perovskite quantum dots, surface ligand, amino acids, excited state dynamics, white light-emitting diode

## Abstract

Organic–inorganic hybrid perovskite quantum dots (QDs) have garnered significant research interest owing to their unique structure and optoelectronic properties. However, their poor optical performance in ambient air remains a significant limitation, hindering their advancement and practical applications. Herein, three amino acids (valine, threonine and cysteine) were chosen as surface ligands to successfully prepare highly luminescent CH_3_NH_3_PbBr_3_ (MAPbBr_3_) QDs. The morphology and XRD results suggest that the inclusion of the amino acid ligands enhances the octahedral structure of the QD solutions. Moreover, the observed blue-shifted phenomenon in the photoluminescence (PL) aligns closely with the blue-shifted phenomenon observed in the ultraviolet–visible (UV-Vis) absorption spectra, attributed to the quantum confinement effect. The time-resolved spectra indicated that the introduction of the amino acid ligands successfully suppressed non-radiative recombination, consequently extending the fluorescence lifetime of the MAPbBr_3_ QDs. The photoluminescence quantum yields (PLQYs) of the amino acid-treated MAPbBr_3_ QDs are increased by 94.8%. The color rendering index (CRI) of the produced white light-emitting diode (WLED) is 85.3, with a correlated color temperature (CCT) of 5453 K. Our study presents a novel approach to enhancing the performance of perovskite QDs by employing specially designed surface ligands for surface passivation.

## 1. Introduction

Perovskite QDs have attracted widespread research interest in the field of optoelectronic devices due to their excellent photonic properties [1,2,3], including their tunable bandgap, high optical absorption coefficient, high defect tolerance and high fluorescence quantum yield. The unique structural properties of perovskite QDs endow them with outstanding optoelectronic performance, rendering them high-performance materials for optoelectronic applications in LEDs, photodetectors and solar cells [4,5,6,7,8]. However, compared to inorganic perovskite QDs, organic–inorganic hybrid perovskite QDs may encounter issues such as a poor optical performance and reduced fluorescence quantum yield due to surface defects, leading to the excessive trapping of free excitons [9,10]. Therefore, significant efforts have been devoted to solving the problems of organic–inorganic hybrid perovskite QDs. For instance, through strategies such as encapsulation with silica, mesoporous silica or polyhedral oligomeric silsesquioxane (POSS), alumina passivation of perovskite QDs via atomic layer deposition have been obtained [11,12]. And protection within hydrophobic phosphate glass and poly(methyl methacrylate) polymers have been pursued. However, such insulating coatings may impede charge transfer between QDs, resulting in the poor conductivity of these embedded perovskite QDs.

Typically, long-chain octylamine (OLA) and oleic acid (OA) are commonly used as traditional ligands in the synthesis process of organic–inorganic hybrid perovskite QDs [13,14]. During growth, the binding between the OLA, OA and the QD surface is relatively loose, achieving a dynamic equilibrium state through continuous dissociation and adsorption. Over time, surface ligands continuously dissociate, reducing QD stability. Furthermore, OLA and OA are insulating long-chain ligands, impeding charge transport and impacting the optoelectronic properties of perovskite QDs [15,16]. To address these issues, efforts have been devoted to developing surface ligand modification methods, known as “ligand exchange”, which has been widely utilized in traditional QD systems [17,18,19,20]. Not all developed methods for ligand modification are suitable for the effective application in halide perovskite QDs, especially those involving the use of polar solvents to transfer short or conductive ligands onto the QD surface. Polar solvents have the potential to easily compromise the structure of halide perovskite QDs, leading to disruption. Additionally, the purification process utilizing polar solvents often causes a significant decline in the optical performance of these QDs [21,22]. Therefore, it is crucial to identify appropriate surface ligands to maintain the stability and optical properties of colloidal perovskite QDs.

Amino acids, being natural molecules, are known as amphoteric ligands, exhibiting a strong affinity with Pb^2+^ ions through their amino groups. In our previous research, we successfully synthesized highly stable MAPbBr_3_ QDs by utilizing L-alanine as a surface ligand, bridging CH_3_NH_3_Br and PbBr_2_ [23]. In this study, we employed three amino acids (valine, threonine and cysteine) to modify the surface of MAPbBr_3_ QDs, demonstrating the utilization of amino acids with diverse structures as surface ligands to fabricate MAPbBr_3_ QDs with enhanced photoluminescence performance. The inclusion of three amino acid ligands decreased the average size of the initial QDs without affecting the cubic structure of the original crystals. Due to the quantum confinement effect, the UV-visible absorption and fluorescence spectra revealed a noticeable blue shift in the absorption and emission peaks of the MAPbBr_3_ QDs upon the introduction of the amino acid ligands. Among them, the introduction of cysteine resulted in a higher PLQY. Additionally, the ultrafast spectroscopy indicated that the introduction of amino acid ligands enhanced the radiative recombination process, thereby increasing the fluorescence lifetime. Based on the excellent optical properties of MAPbBr_3_ QDs modified with cysteine ligands, they were successfully applied in WLED devices.

## 2. Experimentation Section

### 2.1. Materials 

Methylamine (CH_3_NH_2_, 40 wt% in aqueous solution, Aladdin, Shanghai, China), lead bromide (PbBr_2_, 99%, Aladdin), oleylamine (OLA, 80–90%, Aladdin), oleic acid (OA, 85%, Aladdin), toluene (C_7_H_8_, analytical grade, Aladdin), N-N’-dimethylformamide (DMF, 99.9%, analytical grade, Aladdin), hydrobromic acid (HBr, 48%, Aladdin), valine (C_5_H_11_NO_2_, 98%, Tianjin heowns Co., Ltd., Tianjin, China), threonine (C_4_H_9_NO_3_, 97%, Tianjin heowns Co., Ltd.) and cysteine (C_3_H_7_NO_2_S, 98%, Tianjin heowns Co., Ltd.). All materials were used without further purification. 

### 2.2. Synthesis Process of CH_3_NH_3_Br 

A three-necked flask was first pumped to vacuum, and then methylamine (1 mL) and anhydrous ethanol (5 mL) were added to the flask; magnetic stirring was performed to fully mix the reaction solution. A volume of 1.2 mL of hydrobromic acid was slowly added to the flask, and the reaction was stirred for 2 h in an ice bath environment. After the reaction, we used a rotary evaporator to remove the solvent in the reaction system to obtain a crude product. The crude product was washed 3 times with anhydrous ether and dried in a vacuum drying oven for 24 h to obtain the final product, CH_3_NH_3_Br.

### 2.3. Synthesis Process of CH_3_NH_3_PbBr_3_ QDs

In a typical synthesis process, 1.7 mg (0.015 mmol) valine, CH_3_NH_3_Br (9 mg, 0.08 mmol), and PbBr_2_ (36 mg, 0.1 mmol) were added to 1 mL of DMF under magnetic stirring at room temperature. After the solid was completely dissolved, OA (60 μL) and OLA (25 μL) were added to the mixture. Then, we selected three reaction vials and added 40 μL of the three amino acids into each vial; the mixture was stirred for 10 min to obtain a precursor solution. After the reaction, 3 mL of toluene were added into 40 μL of the aforementioned precursor solution separately under magnetic stirring. Once added, the solution immediately turned from yellow to green. A centrifuge was utilized at 7000 rpm for 10 min to separate the four types of QD solutions. The centrifugation process was repeated 3 times and the upper clear liquid was collected to obtain final Val-QDs.

The synthesis process for Thr-QDs and Cys-QDs was identical to that for Val-QDs, with the exception of 1.8 mg (0.015 mmol) of threonine and 1.8 mg (0.015 mmol) of cysteine being used in place of valine.

### 2.4. Fabricating Process of WLED Device 

The Cys-QDs obtained were combined with polyethylpyrrolidone (PVP) to form the composite fluorescent material. Following this, we mixed the commercial blue chip, quantum dots (QDs), and (Sr, Ca) AlSiN_3_:Eu phosphor in a 2:3:1 ratio to form a mixture. The resulting mixture was heated and cured with silica gel, then placed in a vacuum drying oven to remove bubbles. Next, glue was applied using a dispensing machine, followed by the chip being heated at 140 °C for 1 h to produce the WLED device. Subsequently, the photoelectric performance of the device was evaluated.

### 2.5. Characteristic Section

The morphology and the size distribution of the QDs were investigated using a transmission electron microscope (TEM, JEM2800, JEOL Ltd., Tokyo, Japan). The samples for the TEM examination were prepared by directly drying a drop of a dilute isopropanol or toluene dispersion solution containing QDs on the surface of a copper grid. Powder X-ray diffraction (PXRD) data were recorded using a Bruker D2 PHASER diffractometer (Billerica, MA, USA) with Cu Kα radiation and wavelength λ = 1.54056 Å. Fourier transform infrared (FTIR) spectroscopy was performed using a Bruker VERTEX 70 V FTIR spectrometer (Ettlingen, Germany). Absorption spectra of perovskite QDs in the visible and ultraviolet range were measured by UV-Vis spectrophotometer (UV-3600 Plus) (Shimadzu Corporation, Kyoto, Japan). The spectral range was from 300 nm to 600 nm. Steady-state photoluminescence spectra were obtained by a spectrofluorometer FS5 manufactured by Edinburgh Instruments (Livingston, Scotland, UK). The spectral range was from 400 nm to 600 nm. The absolute PLQYs of diluted QD solutions were determined using a fluorescence spectrometer with an integrated sphere (FLS 1000) (Livingston, Scotland, UK) at 365 nm excitation wavelength. The nanosecond transient absorption (ns-TA) was measured with a homemade apparatus based upon a Continuum Surelite II laser (5 ns pulse width, 20 Hz) (Continuum Electro-Optics, Inc., San Jose, CA, USA) providing an excitation wavelength of 532 nm. 

## 3. Results and Discussion

**Figure 1:**
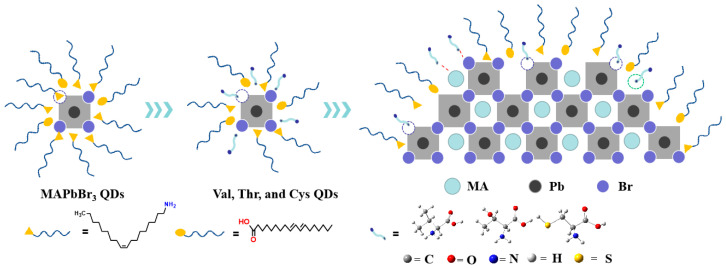
Schematic process for synthesizing MAPbBr_3_ QDs, Val-QDs, Thr-QDs and Cys-QDs.

In our study, we prepared three different amino acid-capped perovskite QDs using a ligand-assisted precipitation method. Figure 1 illustrates the synthetic scheme of the four perovskite QDs. Specifically, we utilized three key amino acids in the synthesis process: valine, threonine and cysteine. These three amino acids have very similar main chain lengths but differ in their substituents: methyl, hydroxyl and thiol groups. Further investigation into the influence of different substituent surface ligands on the optical properties of perovskite QDs was conducted through steady-state and transient spectroscopic characterization.

**Figure 2:**
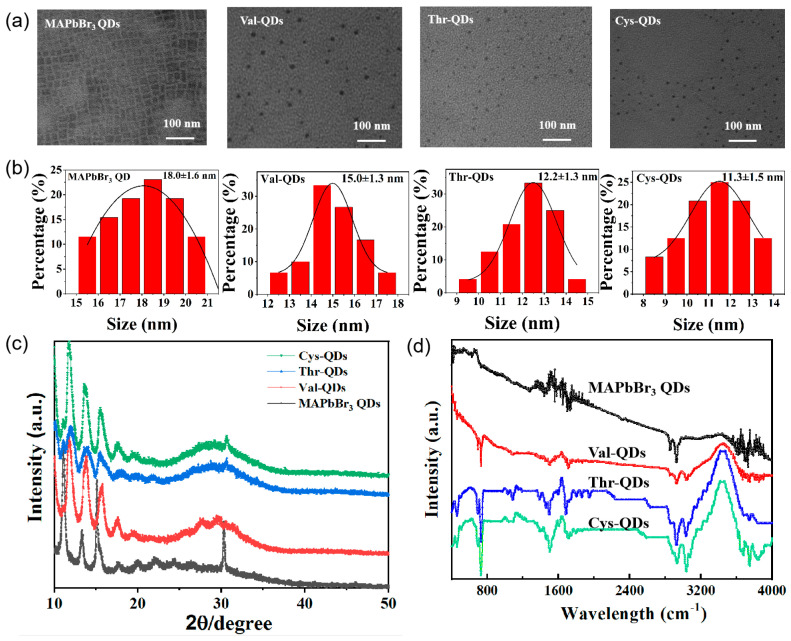
(**a**) TEM, (**b**) particle size distribution, (**c**) XRD diffraction patterns and (**d**) FT- IR spectra of MAPbBr_3_ QDs, Val-QDs, Thr-QDs and Cys-QDs, respectively.

To investigate the surface morphology of MAPbBr_3_ QDs prepared using various amino acid molecules as surface ligands, we utilized TEM testing for characterization [24,25,26]. As depicted in Figure 2a, the TEM images show that the QDs transition from a clear cubic crystal structure to a spherical morphology upon the introduction of amino acid ligands. Meanwhile, the particle size statistics in Figure 2b reveal that as the steric hindrance of the amino acid ligands decreases, the size of the perovskite quantum dots gradually diminishes, and the measured particle sizes were 18.0 ± 1.6 nm, 15.0 ± 1.3 nm, 12.2 ± 1.3 nm, and 11.3 ± 1.5 nm. To delve into the crystal structures of the QDs, we conducted XRD tests on the QDs individually, as depicted in Figure 2c [27,28,29]. The diffraction pattern of the MAPbBr_3_ QDs reveals characteristic peaks at 15.10° and 30.28°, corresponding to the (001) and (002) crystal planes of the cubic phase structure of MAPbBr_3_. This confirms that the synthesized MAPbBr_3_ QDs indeed possess a cubic phase structure. Upon the introduction of amino acids as ligands, the characteristic diffraction peaks around 30° exhibited a decrease in intensity, while the remaining peaks retained their strong characteristics. As shown in Figure 2c, additionally, a series of diffraction peaks at low angles are due to periodic superstructures [30]. Notably, no new distinctive diffraction peaks emerged, affirming that the addition of the amino acids did not alter the cubic structure of the original crystal.

To further understand the interplay between the amino acid ligands and QD surfaces, we conducted infrared spectroscopy analysis on the prepared perovskite QDs [31]. Observing the figure, it is evident that the infrared spectra of the four prepared MAPbBr_3_ QDs exhibit a high degree of consistency. The 1470 cm^−1^ peak in the original MAPbBr_3_ QDs corresponds to the bending vibration of C-H in -CH_3_. Additionally, the two peaks at 2930 cm^−1^ and 2850 cm^−1^ correspond to the stretching vibration of the CH bonds. Moreover, the peak at 1700 cm^−1^ signifies the stretching vibration of the C=O bond within the carboxyl group, while the peak at 1280 cm^−1^ indicates the stretching vibration of the C-O bond within the carboxyl group [32]. Simultaneously, beyond 3600 cm^−1^, the presence of chaotic peaks denotes absorption peaks common to the four prepared MAPbBr_3_ QDs. This observation suggests the interplay between the N-H and O-H absorption peaks, forming distinct characteristic peaks. On the contrary, within the infrared spectrum of Val-QDs, the faint absorption peak at 1080 cm^−1^ aligns with valine’s distinctive absorption peak. Meanwhile, the robust and precise peak at 730 cm^−1^ corresponds to the N-H bond vibration within the amino group. Notably, these absorption peaks manifest similarly in comparable positions within the spectra of the other two MAPbBr_3_ QDs. In the Thr-QD curve, the 1380 cm^−1^ peak aligns with the threonine ligand’s absorption, while the trio of consecutive peaks from 1800 cm^−1^ to 1940 cm^−1^ mirrors threonine’s absorption spectrum. In the curve of the Cys-QDs, the weaker absorption peak split at 1750 cm^−1^ corresponds to the vibration of the S-H bond in cysteine, while the broadening of the absorption peak at 1100 cm^−1^ is also related to cysteine. In summary, the infrared spectrum analysis reveals the successful coupling of the three amino acids (Val, Thr and Cys) to the surface of the MAPbBr_3_ QDs [33,34,35].

**Figure 3:**
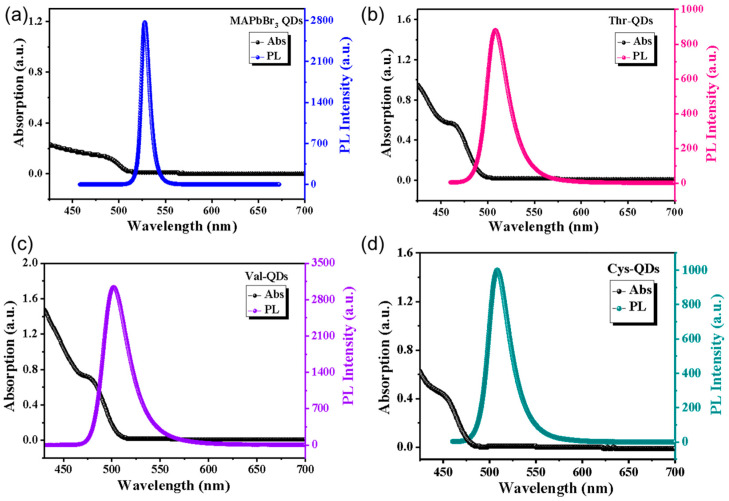
Absorption and emission spectra of (**a**) original MAPbBr_3_ QDs, (**b**) Thr-QDs, (**c**) Val-QDs and (**d**) Cys-QDs.

To further explore the optical properties of the four QDs, we employed UV-Vis absorption spectra and fluorescence spectra to comprehensively characterize their behavior [36,37]. The absorption and emission spectra of the four prepared MAPbBr_3_ QDs are visually presented in Figure 3. As shown in Figure 3, the shoulder peaks observed in the UV-Vis absorption spectrum curve distinctly correspond to the recombination of free excitons [38]. Furthermore, referencing the parameters in Table 1 reveals a noteworthy consistency between the UV-Vis absorption peaks of the four QDs and the respective fluorescence emission peaks. Indeed, the fluorescence luminescence observed in the four QDs is a result of the electrons’ radiative recombination excited to the valence band and the subsequent holes in the conduction band [39,40]. Compared to the original MAPbBr_3_ QDs, those modified with amino acid ligands showcase noticeable blue-shifted absorption peaks. The sizes of the amine acid-modified MAPbBr_3_ QDs become smaller, which is consistent with the above TEM results. Notably, the Cys-QDs demonstrate the most pronounced blue-shifted phenomenon within this set. Additionally, we tested the PLQYs of the original MAPbBr_3_ QDs, Thr-QDs, Val-QDs and Cys-QDs. The values of the four QDs are 86.7%, 90.3%, 91.4% and 94.8%, respectively. Compared to valine and threonine, the sulfur atom in cysteine exhibits a strong coordination ability, which effectively fills the surface defects and reduces non-radiative recombination caused by surface states. Additionally, it enhances the charge transfer efficiency and reduces the recombination probability of electrons and holes, thereby increasing the fluorescence quantum yield. The molecular structure of cysteine also promotes the formation of a compact and ordered ligand layer on the surface of the quantum dots. This ordered layer effectively isolates moisture and oxygen from the environment, enhancing the chemical and optical stability of quantum dots, reducing degradation under light or other environmental conditions, and further increasing the fluorescence quantum yield [41,42].

**Figure 4:**
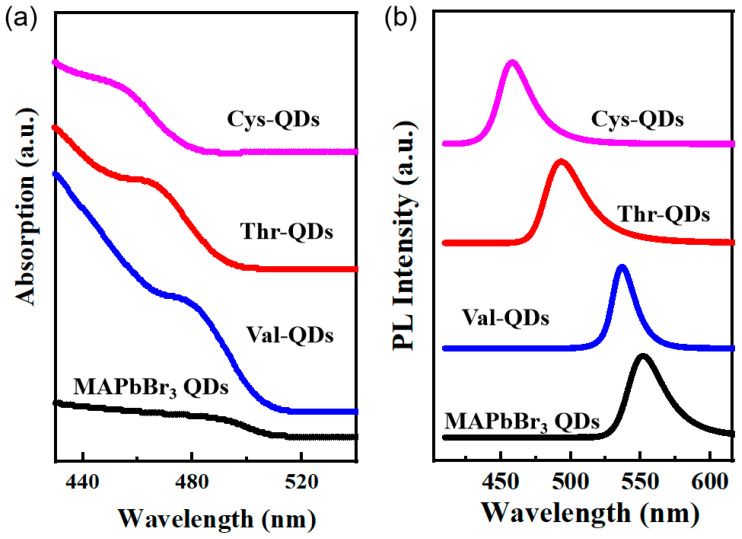
(**a**) UV-Vis absorption spectrum and (**b**) fluorescence spectrum of MAPbBr_3_ QDs, Thr-QDs, Val-QDs and Cys-QDs, respectively.

Figure 4 presents the UV-Vis absorption spectroscopy and PL spectra of the MAPbBr_3_ QDs, Thr-QDs, Val-QDs and Cys-QDs at a 365 nm excitation wavelength. The PL spectrum further demonstrates that the QDs adorned with valine, threonine and cysteine as ligands exhibit a noticeable blue-shifted emission peak. Notably, the most pronounced blue shift persists in the MAPbBr_3_ QDs featuring cysteine as the ligand, aligning consistently with their behavior in UV-Vis absorption spectra. This is attributed to the quantum confinement effect, which demonstrates that when the particle size is reduced to the exciton Bohr radius, it leads to discrete energy level and optical property changes, resulting from the size variation of the MAPbBr_3_ QDs [43]. Moreover, the blue-shifted phenomenon may also be caused by the change in the morphology of the perovskite quantum dots [44]. As the perovskite QDs grow, the introduction of amino acid ligands prompts a reduction in particle size. Consequently, both the absorption and emission peaks of these perovskite QDs undergo a blue-shifted phenomenon influenced by the quantum confinement effect. At the same time, it can be observed that the blue-shifted absorption and emission peaks show a close correlation with the spatial resistance of the amino acid ligands. Notably, the cysteine with the shortest chain length exhibits the least spatial hindrance, yielding QDs of the smallest dimensions. Consequently, these QDs showcase the most significant degree of blue shift in their absorption and emission peaks. The formation of hydrogen bonds within the threonine molecule results in heightened spatial resistance, yielding the largest synthesized QD size, and the least pronounced blue shift in both the absorption and emission peaks of the QDs. These findings align with prior related research [45]. Furthermore, there is a noticeable slight broadening in the emission peak width of the MAPbBr_3_ QDs following the inclusion of the amino acid ligands. This alteration contrasts with the previously observed well-defined symmetry in the emission peak of the original MAPbBr_3_ QDs. Compared to the MAPbBr_3_ quantum dots, the amino acid ligand-modified quantum dots experience a weakening of the emission peak symmetry after photoexcitation. This occurs because some of the electrons that transition to the excited state return to the ground state through non-radiative recombination processes. Therefore, the emission peak symmetry of the other three QDs has diminished. At lower energy levels, the right band tail widens. This occurrence primarily stems from the trapping state capture of a fraction of the surface defects. This enables the generation of fluorescence intensity at these lower energy levels, resulting in tailing and a consequent reduction in the symmetry of the emission peak.

**Figure 5:**
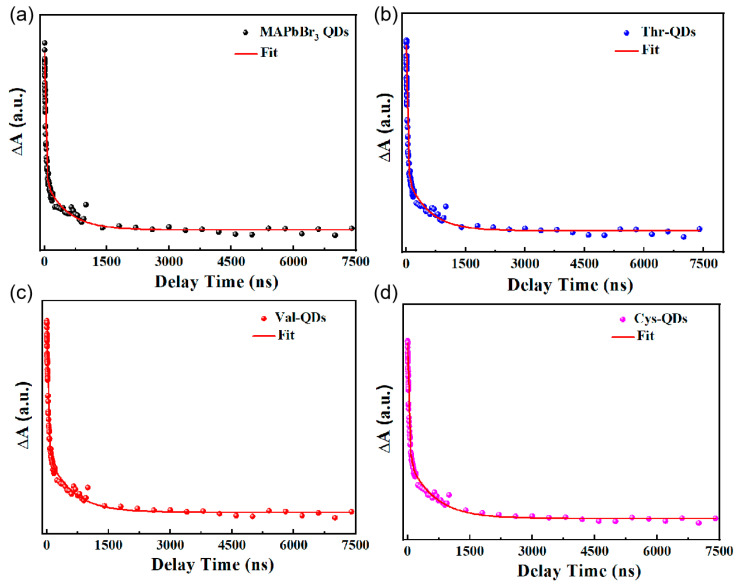
(**a**) Original MAPbBr_3_ QDs, (**b**) Thr-QDs, (**c**) Val-QDs and (**d**) Cys-QDs. Fluorescence decay fitting curves.

Apart from the steady-state spectra characterization mentioned above, we conducted nanosecond transient absorption (ns-TA) spectrum measurements on four QDs to glean insights into the carrier dynamics. The excitation light at a wavelength of 365 nm was employed to excite the four QDs, and the resulting fluorescence decay curves are depicted in Figure 5. The equations for the fitted fluorescence decay curve and the formula for calculating the average lifetime are as follows [46,47]:

(1) $$A=A_{1}\exp\left(-\frac{t}{\tau_{1}}\right)+A_{2}\exp\left(-\frac{t}{\tau_{2}}\right)+A_{0}$$

(2) $$\tau_{\mathrm{avg}}=(A_{1}{\tau_{1}}^{2}+A_{2}{\tau_{2}}^{2})/(A_{1}\tau_{1}+A_{2}\tau_{2})$$

Table 2 presents the pertinent parameters derived from the fluorescence decay fitting curve. Upon analyzing these results, it becomes evident that the fluorescence lifetime of the initial MAPbBr_3_ QDs comprises two distinct components: the fast decay life (fast component) originating from non-radiative recombination and the slow decay life (slow component) arising from radiative recombination. The fast decay lifetime clearly serves as a direct indicator of the surface defect states in the MAPbBr_3_ QDs. Initially, the fast decay lifetime of the original MAPbBr_3_ QDs stands at 25.37 ns. Upon introducing various amino acids as ligands, the respective fast component lifetimes exhibit increments of 29.41 ns, 26.95 ns and 32.28 ns. This increase in the fast component lifetimes signifies an extended duration for defect states to capture free excitons, effectively suppressing non-radiative recombination. Indeed, the proportion of the fast decay lifetime in the original MAPbBr_3_ QDs was measured at 12.8%. Following the addition of amino acids as ligands, this proportion decreased to varying extents. Notably, the fast component constitutes the smallest proportion in the fluorescence lifetime of the Cys-QDs, accounting for only 10.4%. The original MAPbBr_3_ QDs displayed an average lifetime of 475.04 ns. However, after treating the MAPbBr_3_ QDs with three amino acid ligands, there was an increase in the average lifetime. Notably, the Cys-QDs exhibited the longest average lifetime, measuring at 555.49 ns.

The reduction in the proportion of fast components indicates a decrease in defect states and the proportion of non-radiative recombination. This situation allows more free excitons to engage in radiative coincidence, consequently generating fluorescence [48,49]. Thus, a smaller proportion of fast decay lifetime signifies fewer surface defect sites, leading to the more robust suppression of non-radiative recombination and resulting in a heightened radiative luminescence efficiency. Certainly, the comparison among the MAPbBr_3_ QDs treated with three amino acid ligands reveals that the average fluorescence lifetime of the QDs modified with threonine ligands does not show significant improvement. Simultaneously, the proportion of fast components is not notably lower than that of the original MAPbBr_3_ QDs. This phenomenon can be attributed to the side chain substituent of threonine, which includes a hydroxyl group. The oxygen atom within the hydroxyl group engages in intermolecular hydrogen bonding within the solvent, forming connections among short-chain amino acid molecules, thereby creating a long-chain molecule. This increased steric hindrance hampers the effectiveness of other ligands in modifying surface defects, resulting in an insignificant enhancement in surface defect modification. Certainly, the shorter chain length and minimal steric hindrance of cysteine enable it to effectively modify more surface defects, leading to the most pronounced effect among the three amino acids. In summary, all three amino acids possess the ability to enhance the surface dynamics of the MAPbBr_3_ QDs to varying extents. Among them, the Cys-QDs exhibit the most significant degree of modification to the surface defects of the MAPbBr_3_ QDs, while the modification impact of threonine appears less evident.

**Figure 6:**
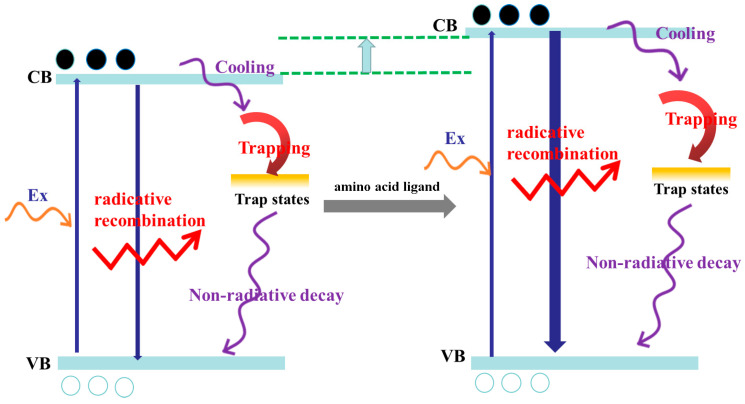
The dynamic mechanism of amino acid-treated MAPbBr_3_ QDs.

Based on the above analysis, we obtained the carrier dynamics mode of the MAPbBr_3_ QDs modified with amino acid ligands, as shown in Figure 6. When the sample is irradiated by 365 nm excitation light, the electrons in the valence band are excited to the excited state. At this time, the valence band will produce holes. Holes and electrons have a strong Coulomb interaction between electron–hole pairs known as carriers. Carriers absorbing higher-intensity pump light will jump to a higher energy level of the excited state, which is in a non-equilibrium state, and they become hot carriers. When the characteristic temperature of the hot carriers is higher than the lattice temperature, the hot carriers will interact with the QD lattice and transfer energy to the lattice to complete cooling. During the cooling process, the carriers will relax to the conduction band and return to the equilibrium state. After reaching the conduction band, the carriers will quickly relax to the edge of the conduction band to form free excitons. There are two ways for free excitons to return to the valence band: the first is radiative recombination; the second is that a part of the exciton is captured in a self-trap state and then returns to the valence band through Auger recombination [50].

The above-described dynamic process will occur in the original MAPbBr_3_ QDs. For the other three amine acid-treated QDs, the carriers will absorb the pump light and transition to a higher energy level of the excited state after adding the amino acids as ligands. This explains why the absorption peaks and emission peaks of the three QDs are blue-shifted. At the same time, after adding the amino acids as ligands, the self-trapped state cannot quickly capture free excitons, and the Auger recombination process is suppressed. This also explains why the values of the three types of QDs in Table 1 have been measured. Since Auger recombination is suppressed, more free excitons at the edge of the conduction band return to the ground state through radiative recombination, which greatly extends the lifetime of the fluorescence emission. Specifically, the average lifetime of the three amine acid-treated QDs is increased. Comparing the three amine acid-treated MAPbBr_3_ QDs, the excited state that the carriers of the Cys-QDs transition into is the highest among those of the amine acid-treated QDs, and in turn shows the highest degree of blue shift in the steady-state spectrum. The excited state that the carriers of the Thr-QDs transition into after absorbing pump light is the lowest among the amine acid-treated QDs, and its steady-state spectrum has the smallest blue shift. The three different amino acids have different effects on the probability of capturing free excitons in the self-trapped state. The addition of cysteine makes it impossible for the self-trapped state to quickly capture free excitons, allowing more free excitons to undergo radiative recombination. This observation highlights that threonine’s fluorescence lifetime is the shortest among the three and constitutes the largest proportion. The addition of threonine did not significantly affect the trapping of the self-trapped excitons, resulting in the shortest fluorescence lifetime and the highest proportion among the three amino acid-treated QDs.

**Figure 7:**
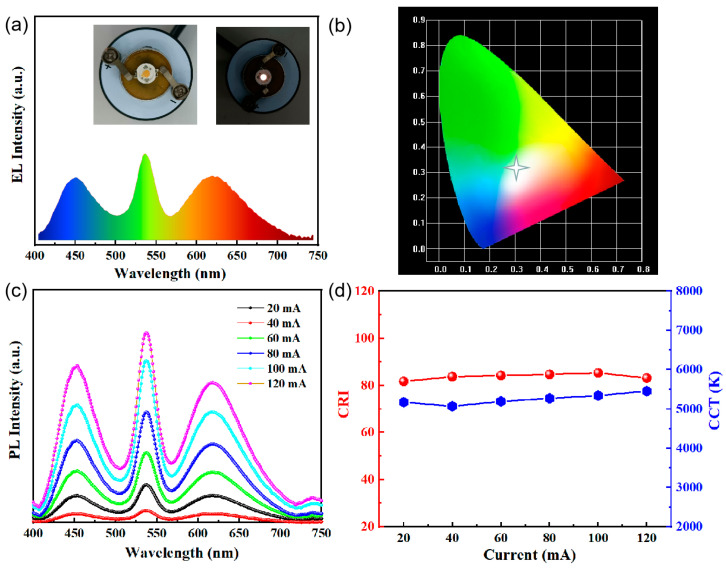
(**a**) EL spectrum and (**b**) CIE coordinate of WLED; (**c**) EL spectra and (**d**) evolution curves of CRI and CCT in different currents.

Leveraging the excellent optical performance of the prepared Cys-QDs, they were successfully applied in WLED devices [51,52]. The WLED was fabricated by encapsulating a mixture of green-emitting Cys-QDs, red-emitting (Sr, Ca) AlSiN_3_:Eu phosphor, and a blue InGaN LED chip. As shown in Figure 7a, the electroluminescence (EL) spectrum comprises three emission bands centered at 453 nm, 500 nm and 624 nm, corresponding to the blue LED chip, Cys-QDs and (Sr, Ca) AlSiN_3_:Eu phosphor, respectively. Additionally, Figure 7c illustrates the optoelectronic performance of the WLED under various forward currents. To study the effect of changing the LED current on the CRI and CCT, the WLED device drive current was increased from 20 mA to 120 mA, the EL intensity was gradually increased, the color rendering index (CRI) was raised from 81.7 to 85.3, and the correlated color temperature (CCT) was increased from 5172 K to 5453 K (Figure 7d). By adjusting the current, we can observe that the LED maintains its performance in terms of light quality and color characteristics.

## 4. Conclusions

In summary, we successfully synthesized high-performance MAPbBr_3_ QDs utilizing various amino acids (valine, threonine and cysteine) as surface ligands. The FTIR results initially confirmed the successful bonding of all three amino acids to the QD surfaces. The subsequent XRD testing revealed that while the introduction of these amino acids as surface ligands reduced the size of the QDs, it did not alter their original cubic structure. Due to the quantum confinement effect, the MAPbBr_3_ QDs protected by amino acid ligands exhibit a blue-shifted phenomenon in their absorption and emission peaks towards shorter wavelengths. The time-resolved ultrafast spectroscopy unveiled a remarkable reduction in surface non-radiative recombination in the MAPbBr_3_ QDs adorned with valine and cysteine amino acid ligands, facilitating a greater number of free excitons to engage in radiative recombination and emit light. However, the impact of the threonine amino acid on surface defect modification was less pronounced, and this is attributed to the hydroxyl groups on its side chain forming hydrogen bonds, which led to excessive steric hindrance and fewer alterations of surface defects. Compared with the pure MAPbBr_3_ QDs, those modified with cysteine as a surface ligand exhibit a significantly enhanced performance. WLED was successfully fabricated using the Cys-QDs and red nitride composite phosphor as well as a blue chip with stable CRI and CCT values. This work has achieved significant breakthroughs in surface ligand regulation, paving the way for the development of innovative hybrid solar cells, LED semiconductors, and other advanced materials.

## Figures and Tables

**Table 1:** Absorption and emission parameters of original MAPbBr_3_ QDs, Thr-QDs, Val-QDs and Cys-QDs.

	MAPbBr_3_ QDs	Val QDs	Thr QDs	Cys QDs
Absorption wavelength (nm)	490	480	470	460
Emission wavelength (nm)	550	534	515	500

**Table 2:** Fluorescence decay fitting curve parameters of original MAPbBr_3_ QDs, Thr-QDs, Val-QDs and Cys-QDs.

Sample	$\tau_{1\;}$(ns)	$\tau_{2}$ (ns)	*A*_1_ (%)	*A*_2_ (%)	$\tau_{avg}$ (ns)
MAPbBr_3_ QDs	25.37	541.26	12.8	87.2	475.04
Val-QDs	29.41	580.23	11.2	88.8	518.28
Thr-QDs	26.95	547.55	12.3	87.7	483.84
Cys-QDs	32.28	616.29	10.4	89.6	555.49

## Data Availability

Data will be made available on request.
